# Reduced blood pressure variability in AS COT -BPLA trial favours use of amlodipine/perindopril combination to reduce stroke risk

**Published:** 2010-04

**Authors:** J Aalbers

**Affiliations:** Special Assignments Editor

Lower within-individual visit-to-visit variability in blood pressure readings in the amlodipine/perindopril combination treatment arm of the ASCOT study has been shown to account for the more effective stroke reduction rate with this combination compared to atenolol and diuretic-based therapies, despite overall similar blood pressure lowering.

This finding was presented at the late-breaking clinical trial session of the American College of Cardiology congress and was published simultaneously in the Lancet Neurology edition online.^1^

‘These findings have major clinical implications for the management of patients with hypertension’, said Peter Sever, FRCP, a professor of clinical pharmacology and therapeutics and co-director of the International Centre for Circulatory Health, Imperial College, London. ‘This data convincingly demonstrates that patients with more variation in their blood pressure levels are at greatest risk of future heart attacks and strokes, and that reducing variability is a key goal of treatment.’^2^

For the ASCOT-BPLA study, researchers recruited 19 257 patients with high blood pressure, averaging 164/95 mmHg at rest. Patients were randomly assigned to treatment with the calcium channel blocker amlodipine with or without the ACE inhibitor perindopril, or to treatment with the beta-blocker atenolol with or without the diuretic bendroflumethiazide.During 5.5 years of follow up, blood pressure variability was determined by comparing multiple blood pressure readings taken at each visit and at several different visits.

The Medical Research Council (MRC) trial of 4 396 elderly hypertensive patients treated with atenolol, a diuretic combination, or placebo, were also evaluated for blood pressure variability and the results were included in this study.^3^

Researchers found that between-visit blood pressure variability was significantly greater among patients treated in the atenolol arm than those in the amlodipine/perindopril arm of ASCOT. In addition, when researchers compared patients at the highest one-tenth for between-visit blood pressure variability to those at the lowest one-tenth, they found a strong link with increased risk of stroke.

In the beta-blocker group, patients with high blood pressure variability faced a risk of stroke 4.06 times that of those with low blood pressure variability. In the amlodipine/perindopril group, the risk of stroke was 3.8 times higher among patients with high blood pressure variability.

A similar pattern linked high blood pressure variability to an increased risk for heart attack and other coronary events. The overall risk of stroke was 22% lower among patients treated with amlodipine/ perindopril when compared to those treated with the beta blocker/thiazide diuretic, a difference that could be entirely explained by differences in blood pressure variability, researchers found.

In the ASCOT-BPLA trial, the researchers were also able to look at within-visit variability and variability on 24-hour ambulatory blood pressure monitoring (ABPM). In the ABPM substudy, reduced variability in daytime systolic blood pressure (SBP) in the amlodipine group (p < 0.0001) partly accounted for the reduced risk of vascular events, but reduced visit-to-visit variability in clinic SBP had a greater effect.

In the MRC trial, all measures of within-individual visit-to-visit variability in SBP were increased in the atenolol group compared with both the placebo and diuretic groups during initial follow up (all p < 0.0001). Subsequent temporal trends in variability in blood pressure during follow up in the atenolol group correlated with trends in stroke risk.

‘The greater reduction in variability among patients treated with amlodipine and perindopril may be best explained by their more profound effects on blood vessel relaxation, compared to beta-blockers’, Sever said. He and his co-investigators believe that clinical guidelines and future drug trials must take into account the importance of blood pressure variability in the management of patients, not simply the extent to which a drug lowers average levels of blood pressure. This aspect will need to be reported on in more detail in future trials.

The hypothesis that erratic blood pressure levels could be an important factor in determining the risk of future strokes was originally proposed by Peter M Rothwell, MD, PhD, of the Stroke Prevention Research Unit, University Department of Clinical Neurology, John Radcliffe Hospital, Oxford, UK.
